# Morphological Signatures of Neurogenesis and Neuronal Migration in Hypothalamic Vasopressinergic Magnocellular Nuclei of the Adult Rat

**DOI:** 10.3390/ijms25136988

**Published:** 2024-06-26

**Authors:** Limei Zhang, Mario A. Zetter, Vito S. Hernández, Oscar R. Hernández-Pérez, Fernando Jáuregui-Huerta, Quirin Krabichler, Valery Grinevich

**Affiliations:** 1 Department of Physiology, School of Medicine, National Autonomous University of Mexico, Mexico City 04510, Mexico; mario.zetter@lasalle.mx (M.A.Z.); vitohdez@unam.mx (V.S.H.); ohernandez@facmed.unam.mx (O.R.H.-P.); 2 Section on Molecular Neuroscience, National Institute of Mental Health, NIH, Bethesda, MD 20892, USA; 3 Department of Medicine and Health, University of La Salle, Mexico City 14000, Mexico; 4 Department of Neuropeptide Research in Psychiatry, Central Institute of Mental Health, Medical Faculty Mannheim, University of Heidelberg, 69120 Mannheim, Germany; quirin.krabichler@zi-mannheim.de (Q.K.); valery.grinevich@zi-mannheim.de (V.G.)

**Keywords:** vasopressin, hypothalamus, neurogenic niche, radial glial cells, microglia, doublecortin (DCX), BrdU, Ki67

## Abstract

The arginine vasopressin (AVP)-magnocellular neurosecretory system (AVPMNS) in the hypothalamus plays a critical role in homeostatic regulation as well as in allostatic motivational behaviors. However, it remains unclear whether adult neurogenesis exists in the AVPMNS. By using immunoreaction against AVP, neurophysin II, glial fibrillar acidic protein (GFAP), cell division marker (Ki67), migrating neuroblast markers (doublecortin, DCX), microglial marker (Ionized calcium binding adaptor molecule 1, Iba1), and 5′-bromo-2′-deoxyuridine (BrdU), we report morphological evidence that low-rate neurogenesis and migration occur in adult AVPMNS in the rat hypothalamus. Tangential AVP/GFAP migration routes and AVP/DCX neuronal chains as well as ascending AVP axonal scaffolds were observed. Chronic water deprivation significantly increased the BrdU+ nuclei within both the supraaoptic (SON) and paraventricular (PVN) nuclei. These findings raise new questions about AVPMNS’s potential hormonal role for brain physiological adaptation across the lifespan, with possible involvement in coping with homeostatic adversities.

## 1. Introduction

Adult neurogenesis is defined as the ability of specialized cells in the postnatal brain to produce new functional neurons. This phenomenon was first formally reported six decades ago using intraperitoneal injection of thymidine-H^3^ in rats and cats, which shed some light on the possibility that new neurons may be formed in forebrain structures in both rodents and carnivores, which was not considered possible previously [1,2,3]. Today, it is well established that the subventricular zone of the lateral ventricles and the subgranular zone of the hippocampus are prominent regions of the neurogenesis that occurs through the lifespan [4,5,6,7,8]. However, the phenomenon has been described in a large number of additional brain regions [9]. For example, adult neurogenesis has also been observed in the striatum of humans [10], in the hippocampal fissure region in rats under pilocarpine insult [11], and in the caudate nucleus and nucleus accumbens in rats [12]. Furthermore, it has been reported in the hypothalamus of several rodent and primate species [13,14,15,16,17,18,19], suggesting that adult hypothalamic neurogenesis may serve as a mechanism for brain physiological adaptation across the lifespan, with possible roles in coping with homeostatic adversities. However, the actual sites and organization of distinct neurogenic niches in the hypothalamus remain elusive.

The hypothalamus hosts unique magnocellular neurosecretory neurons (MCNs), which synthesize the neurohormone arginine vasopressin (AVP) and its homologue oxytocin (OT) [20,21]. Three loci of AVP-MCNs have been described, i.e., the supraoptic (SON), paraventricular (PVN), and accessory (AN) nuclei [22]. Most, if not all, of the AVP-MCNs located within the SON, PVN, and AN emit main axons carrying AVP to the pituitary gland, via the hypothalamic-neurohypophysial tract [23], which exerts control over body water and electrolyte homeostasis, blood pressure, and hepatic glucose metabolism [22,24,25,26]. The role of AVP-MCNs in the hormonal regulation of peripheral functions has been gradually established over the past century. However, a novel understanding of AVP-MCN involvement in higher brain functions, such as memory processes, emotion, and motivation, complementary to their peripheral physiological role, has emerged in recent years (for reviews, see [27,28]).

In the early 1990s, Swaab and colleagues reported that the hypothalamic AVP and OT nuclei change in size and cell number at various postnatal developmental stages in the pig [29,30,31]. These changes were also observed upon alteration of gonadal-reproductive status [32]. The above observations were further confirmed by a Canadian group [33,34], who, by using double-immunohistochemistry against proliferating-cell nuclear antigen (PCNA, a key factor in DNA replication) and either AVP or OT, found evidence suggesting that AVP or OT neurons could undergo postnatal cell division according to physiological demands, such as the commencement of sexual maturity and lactation. While these observations went largely overlooked, it was independently reported in 2004 that progenitor cells could be isolated from the adult rat hypothalamus, which readily differentiated into mature neurons expressing various neuropeptides, among them AVP and OT [35]. However, little is known about the phenomenon of AVP or OT neurons generated by neurogenesis in the adult brain with respect to both neuroanatomical organization and potential functions.

To test the hypothesis that the AVP system could undergo neurogenesis and neuronal dispersion from adult rat hypothalamic magnocellular loci, we conducted a systematic histological examination of serial rat brain sections in the coronal, sagittal, horizontal, and septo-temporal oblique orientations from male and female, juvenile to old (18 months) rats. Here, we report evidence that strongly suggests that some AVP neurons are generated by adult neurogenesis and migrate following blood vessels, glial processes, and axons of other AVP neurons. We challenged rats with chronic water deprivation every other day and administered for two weeks intraperitoneal 5-bromo-2′-deoxyuridine (BrdU), a synthetic nucleoside analogue with a chemical structure similar to thymidine, commonly used to assess cell proliferation in living tissues [1,3,36]. Immunohistochemical analysis revealed BrdU-immunoreactive (ir) large round-shaped nuclei inside the AVP magnocellular loci, with some of these paired as twin BrdU-ir nuclei, indicating ongoing neurogenesis in these areas. We also assessed the co-expression of AVP and the migrating neuroblast marker doublecortin (DCX) and found numerous DCX neurons within the SON and PVN, with some of these also expressing AVP. Our data suggest that AVP neurons in rat hypothalamic SON and PVN may undergo low-rate neurogenesis during adult life and that some of them may migrate to adjacent subcortical regions, as evidenced by the presence of AVP cell chains, which might be guided by axon scaffolds.

## 2. Results

**AVP-ir SON and PVN dispersed to other subcortical regions: DCX-immunopositive migrating cell chains guided by axon scaffolds**. In order to describe the morphology of AVP immunoreactivity in the rat brain, we analyzed serial sections cut in coronal, sagittal, horizontal, and septo-temporal (oblique) orientations. The immunohistochemical reactions were performed in every third sequential section, and we used a combination of two rabbit anti-vasopressin antibodies (gift from Prof. Ruud Buijs [37] and Peninsula laboratories international, T4563). The rationale for the use of a combination of two anti-vasopressin antibodies was to enhance phenotypic characterization by optimizing labeling of the AVP system across changes in the molecular form of vasopressin as prohormone processing ensues.

**Figure 1:**
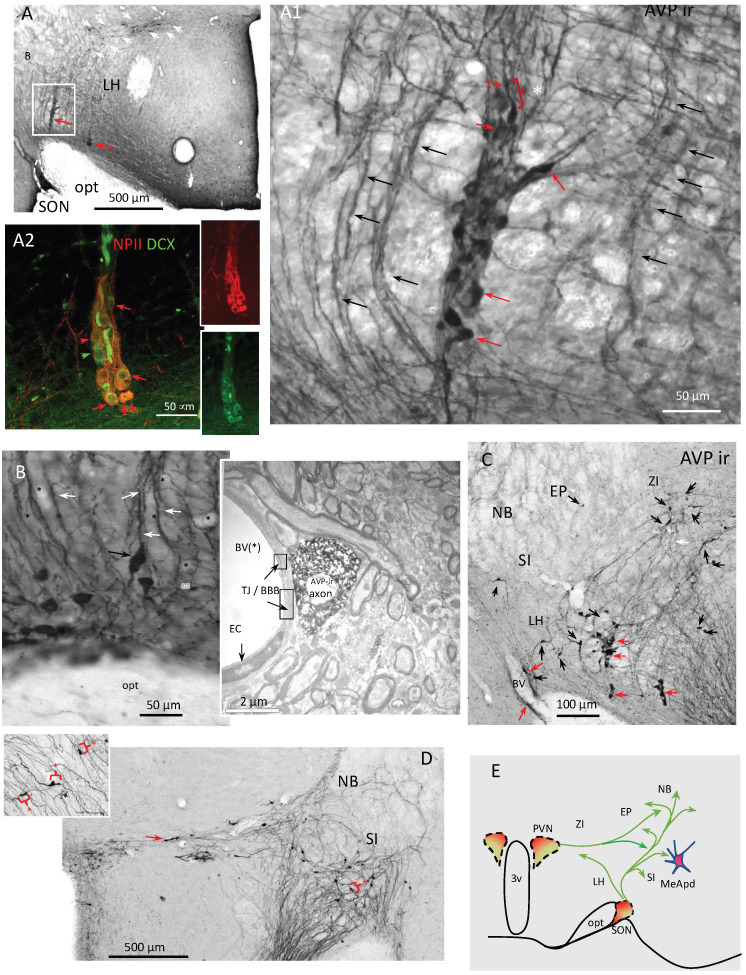
Network of AVP-ir cell chains and axon scaffolds in the adult rat hypothalamus (1/5) emerging from the supraoptic nucleus (SON)—coronal view through serial sections from an adult (4-month-old) male Wistar rat. (**A**,**A1**): AVP-ir cell-chains (red arrows) and axonal scaffolds (black arrows) emerged from SON^AVP^. AVP-ir cells (red arrows) showed tangential migration morphology that is mainly bipolar, with leading process extension and swelling formation for eventual somal translocation [38] (white asterisk and bracket show a cell with somal translocation, as an example). The cell chains were previously characterized as neuronal precursors in migration [7]. The cell chain (**A2**) showed co-expression of neuroblast marker doublecortin (DCX, green) and neurophysin II (NPII, red), a carrier protein for vasopressin. The red arrows indicate the double-expression of NPII and DCX and the green arrow indicates a cell which does not express NPII. Panel (**B**) shows the AVP-ir axonal scaffolds (white arrows). An AVP-ir cell with axon swelling and somal translocation phenotype (black arrow) seemed to “climb” into the AVP-ir scaffolds. The proximity of the AVP-ir axon with blood vessels (indicated by black *) was frequently observed. A transmission electron microscopical picture with AVP IHC reaction taken from a preoptic area of the rat hypothalamus is shown as (**B**) inset. Tight junctions (TJs) of the brain–blood barrier (BBB) formed by processes of endothelium cells (EC) are indicated. The AVP-ir axons are separated from the lumen of blood vessels at the ultra-structural level. (**C**) Presence of AVP-ir cells (black arrows) with tangential migration morphology in entopeduncular nucleus (EP) and lateral hypothalamus (LH). Frequently observed cell chains are indicated with red arrows. The white arrow shows a example of axon scafold. (**D**) AVP-ir cell chain (red arrow) leaving the PVN (red arrow) with lateral-tangential movement morphology. Substantia innominata (SI) and nucleus basalis of Meynert (NB) are innervated with AVP axon scaffolds and disperse AVP-ir cells. Inset shows bipolar neurons with swelling formation for eventual somal translocation, indicated with red asterisk and brackets. (**E**) Charting symbolizing the observed migration routes from coronal samples (green arrows). Abbreviations, NB: nucleus basalis of Meynert; ZI: zona incerta; 3V: third ventricle.

**Figure 2:**
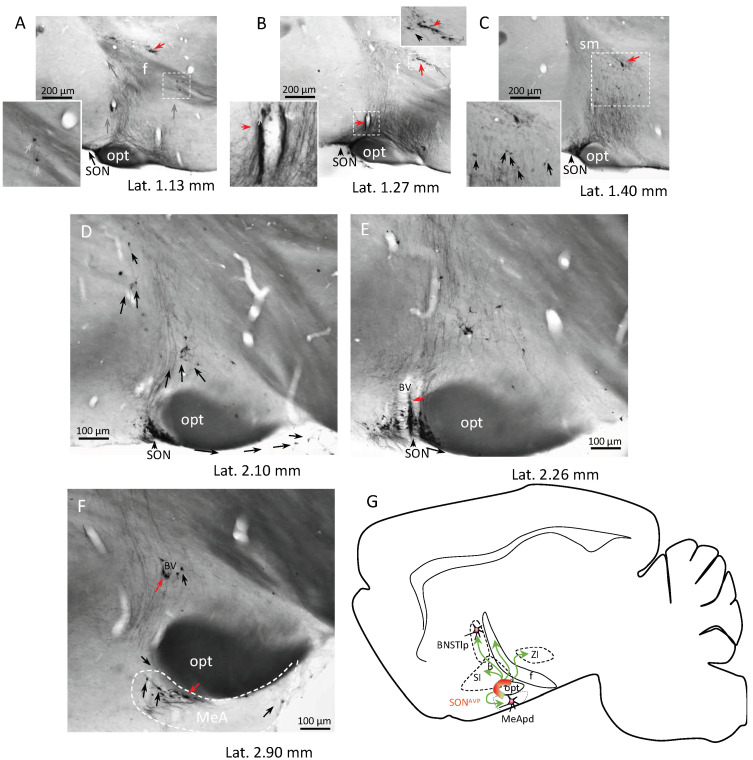
Network of AVP-ir cell chains and axon scaffolds in the adult rat hypothalamus (2/5) emerging from the supraoptic nucleus (SON)—sagittal view through serial sections from an adult female 4-month-old Wistar rat. (**A**–**F**) AVP immunostaining at representative medio-lateral levels. Ascending axonal stream can be clearly identified. AVP-ir cell chains are indicated by red arrows. Dispersed neurons with tangential migration morphology, climbing in the AVP-ir axonal scaffold, are indicated by black arrows. (**D**–**F**) show the continuity and lateral migration path of AVP-ir cells from SON to the medial amygdala alongside the optical tract (opt). (**G**) Charting symbolizing the migration routes (green arrows) visualized from sagittal series analysis. Gray arrows symbolize the apparent migration direction. Samples were osmicated for electron microscopy study because the myelinated fibers were dark. Abbreviations, B: nucleus basalis of Meynert; BSTlp: bed nucleus of stria terminalis, lateral posterior; f: fornix, MeApd: medial amygdala posterodorsal; SI: sustantia innominata; ZI: zona incerta.

**Figure 3:**
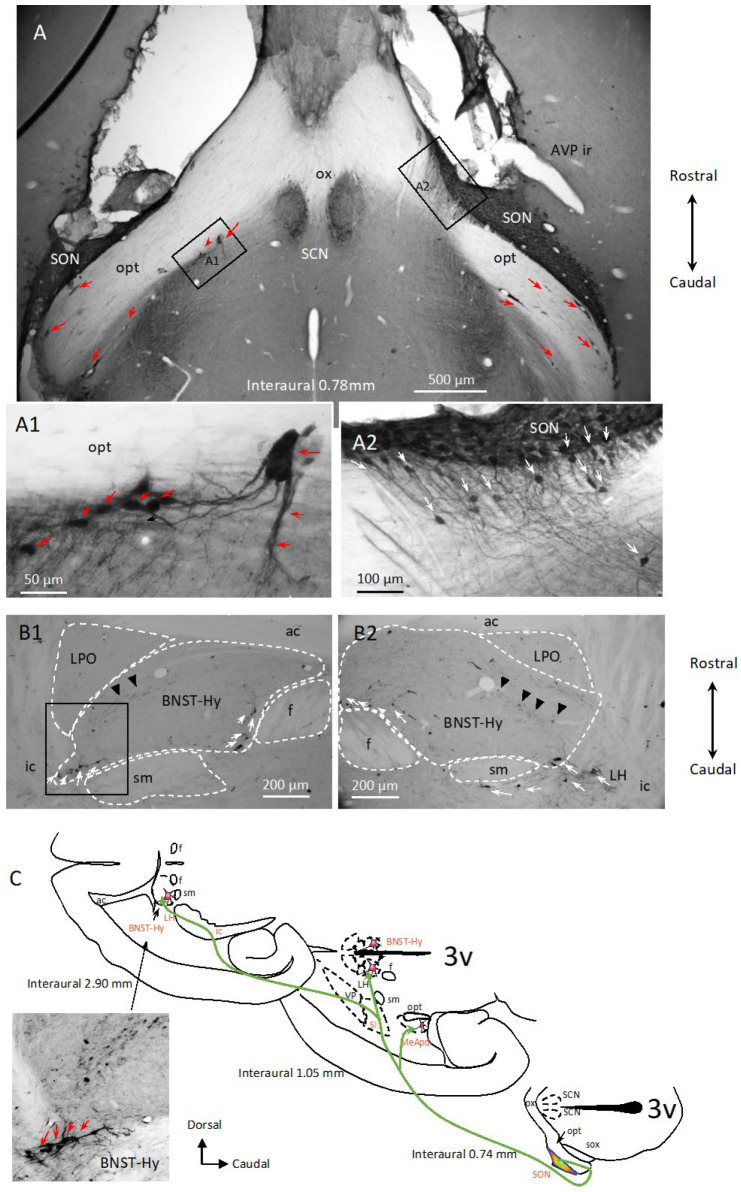
Network of AVP-ir cell chains and axon scaffolds in the adult rat hypothalamus (3/5) emerging from the supraoptic nucleus (SON)—horizontal view through serial sections from an adult male 4-month-old Wistar rat. (**A**,**A1**,**A2**): immunostaining at the interaural level 0.78 mm, where the SON and suprachiasmatic nucleus (SCN) vasopressinergic cells can be clearly seen, but with different features. While the SCN^AVP^ are well delineated, some neurons of SON^AVP^ dispersed tangentially following the axonal scaffolds (**A1**,**A2**), mainly alongside the optic tract (opt). Numerous chains are indicated with red arrows. (**B1**,**B2**) show the tangentially migrated AVP-ir cells arriving the lateral portion of the bed nucleus of the stria terminalis (white arrows). One population of AVP-ir cells is located in the rostral part, with small somata and weak AVP immunostaining (indicated by black arrowheads), which can be clearly distinguished from the population migrated along the stria medullaris and the fimbria. The latter ones are strongly immunoreactive to AVP (white arrows). (**C**) charting symbolizing the migration routes (green arrows) from SON to medial amygdala posterodorsal division (MeApd) and the bed nucleus of the stria terminalis hypothalamus (BNST-Hy), passing through the sustantia innominata (SI), ventral pallidum (VP), land ateral hypothalamus (LH) in 3 horizontal levels visualized by horizontal section analysis.

**Figure 4:**
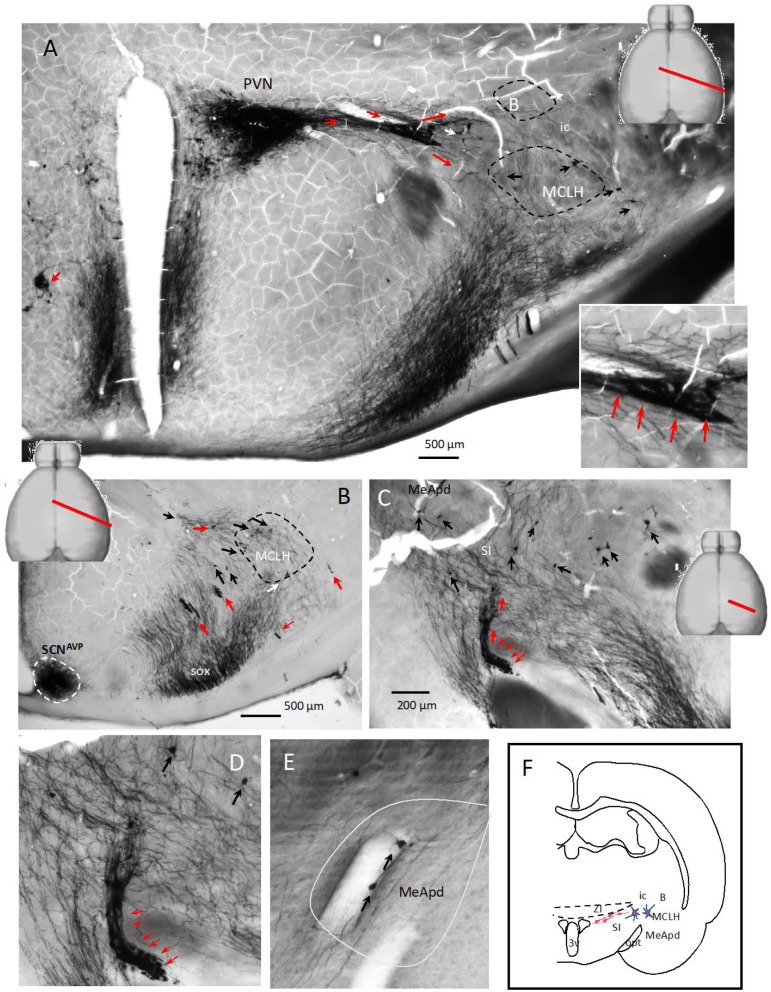
Network of AVP-ir cell chains and axon scaffolds in the adult rat hypothalamus (4/5) emerging from the paraventricular (PVN) and supraoptic nucleus (SON)—septo-temporal oblique view (−30°) through serial sections from a 12-month-old adult male Wistar rat. (**A**): AVP-ir of a septo-temporal oblique cutting plane passing through the center of the PVN with −30 degrees inclination toward the temporo-caudal direction revealed thick a AVP-ir cell chain emerging from the PVN alongside a thick blood vessel, which bifurcated the latero-dorsal. (**B**): Nucleus basalis of Meynert and latero-ventral (MCLH: magnocellular nucleus of the lateral hypothalamus) directions. Red arrows indicate cell chains, and black arrows indicate the dispersed cells. (**B**): More examples of dispersed cells intermingled with axonal scaffolds. Note that the suprachiasmatic nucleus (SCN^AVP^) shows the distinct feature that no cell chains or dispersed neurons are observed to emerge from it (outlined with white dashed line). (**C**): More examples of AVP-ir cell chains and dispersed neurons, with the amplified cell chain shown in (**D**). Panel (**E**) shows tangential migrating AVP-ir cells in the medial amygdala, postero-dorsal subdivision. A bipolar AVP cell alongside a blood vessel, with axonal swelling and apparent somal translocation, is indicated with a black arrow. (**F**): Charting synthesizing the observation of this figure. Samples were osmicated for electron microscopy study because the myelinated fibers were dark.

**Figure 5:**
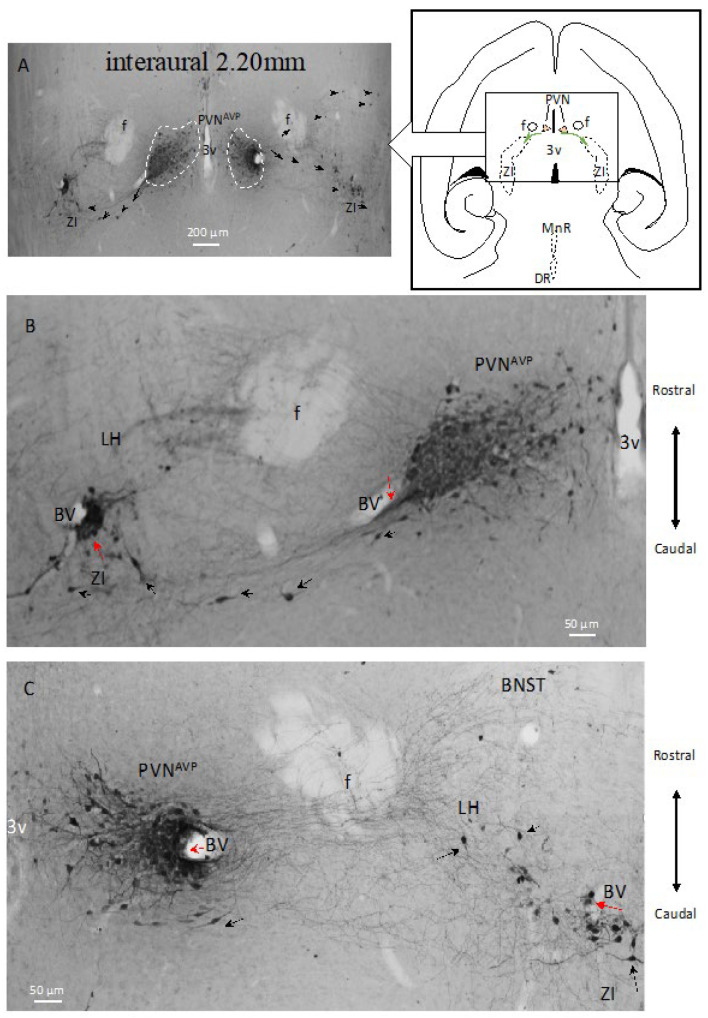
Network of AVP-ir cell chains and axon scaffolds in the adult rat hypothalamus emerging (5/5) from the paraventricular nucleus (PVN)—horizontal view reveals migration route for direct connection between PVN and ZI AVP-ir cells (sections from an adult male 4-month-old Wistar rat). (**A**) AVP-ir cell-chains (red arrows) and axonal scaffolds (indicated by black arrows) emerging from PVN^AVP^ (green arrows symbolize the dispersion direction).^.^ (**B**,**C**) Amplifications of each side of (**A**).

**Figure A1:**
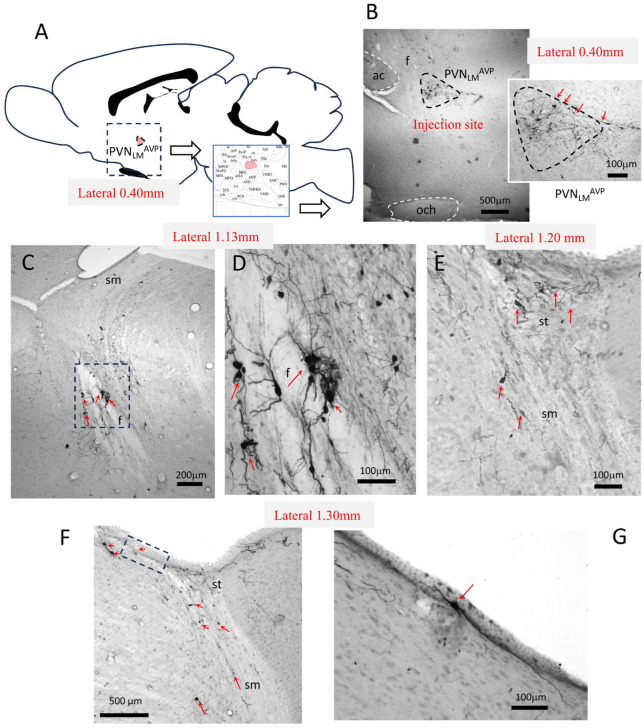
(**A**) and inset: Atlas sagittal showing the location of the lateral magnocellular division of the paraventricular nucleus of the hypothalamus (PVN_LM,_ pink shadow region), which was infected by Cre-dependent AAV equipped with mCherry in an adult male AVP-IRES-Cre knock-in rat. The rat brain was fixed after one month of the viral infection. Panel (**B**) and inset show the injection site with immunohistochemically (IHC)-labeled cells (IHC against mCherry driven by the AVP promoter) within the PVN_LM_^AV^ (outlined with dashed line). The labeling was sparse and precise without positive cells outside of the PVN_LM._ Numerous neurons with tangential migration-like morphology (bipolar) within the PVN_LM_^AVP^ were observed (indicated with red arrows). (**C**): Photomicrograph taken from sagittal section at around lateral 1.13 mm (Paxinos and Watson, 1997): numerous IHC-labeled cells forming cellular chains within the brain’s two main conducting systems, the fornix (f) and the stria medullaris (sm). The squared region was further enlarged in (**D**), showing labeled cells with tangential migration-like morphology (red arrows). (**E**): Photomicrograph taken from sagittal section at around lateral 1.20 mm, showing putatively migrating cells present in another major conducting system, the stria terminalis (st), with migration-like morphology pointed out by red arrows. (**F**) An additional example of the presence of the labeled cells in the sm and adjacent regions, with one bipolar neuron (squared region) in close proximity to the ependyma of the third ventricle (3v) with more than 1 mm of its longitudinal axis, enlarged in (**G**).

Selective planes that could best illustrate the phenomenon of adult neuronal tangential migrations occurring in AVP-ir populations are presented in Figure 1, Figure 2, Figure 3, Figure 4 and Figure 5 and Figure A1, with detailed description in the figure legends. The sex and age of animals from which the series were obtained are also indicated in the legends.

Figure 1 shows examples from the rat brain coronal section series with an emphasis on the supraoptic nucleus (SON). AVP-ir cell chains emerging from the SON can be clearly seen (Figure 1(A,A1)). Their peculiar formation and morphology first led us to hypothesize that they are putative neuronal precursors (indicated with red arrows). Indeed, cells in chains are classical identifying features for neuronal precursors in migration [7]. The AVP-ir cells showed tangential migration morphology that is mainly bipolar, with leading process extension and swelling formation for eventual somal translocation [38] (for instance, the cell indicated with a red asterisk and bracket in A1). Importantly, these cell chains (A2) showed co-expression of neurophysin II (NPII, the carrier protein for AVP; in red) and the neuroblast marker doublecortin (DCX, green). Figure 1B shows the AVP-ir axonal scaffolds with AVP-ir cells putatively climbing along them. The AVP-ir axons in this region (preoptic area of the rat hypothalamus) are often found in close proximity with blood vessels, as shown in the electron photomicrograph Figure 1B—inset. Tight junctions (TJs) formed by processes of endothelium cells can be clearly seen, which indicates that the AVP-ir axons are separated from the lumen of blood vessels by cells of the blood–brain barrier. AVP-ir neurons with the tangential migrating phenotype were further observed in the entopeduncular nucleus (EP; Figure 1C, red arrows), again putatively climbing along AVP-ir axon scaffolds (Figure 1C, black arrows). Figure 1D shows the AVP-ir cell chain leaving the PVN (red arrow) with lateral-tangential movement morphology. The substantia innominata (SI) and nucleus basalis of Meynert (B) are innervated with AVP axon scaffolds and disperse AVP-ir cells. The inset shows bipolar neurons with swelling formation for eventual somatic translocation, indicated with red asterisk and brackets. Through systematic microscopical examination and analysis of these series, we conclude that the AVP-ir neurons could take several routes to migrate from the SON and PVN, with some of them moving dorsomedially towards zona incerta (ZI), but most of them streaming dorsolaterally towards the entopeduncular nucleus (EP), the posterodorsal part of the medial amygdala (MeApd), and the nucleus basalis of Meynert (B) (Figure 1D, green arrows).

The sagittal series of AVP immunostaining revealed massive ascending axonal routes toward the brain main conducting systems, i.e., the fornix and stria medullaris (Figure 2). Numerous cell chains are discovered from this view (Figure 2A–C,E,F, red arrows). AVP-ir cells with tangentially migrating morphology (*vide supra*) were found in adjacent regions to the SON and PVN. Populational continuity between the SON^AVP^ and MeApd^AVP^ can be appreciated through serial section viewing (Figure 2A–F).

The horizontal section series of AVP-ir allowed us to appreciate that many AVP-ir neurons disperse from the SON, alongside the optic tract (opt), forming cell chains (Figure 3A; note that the majority of the cell chains within the optic tract are associated with a blood vessel within the chain, indicated by red arrows). Other AVP-ir cells were dispersed by tangential migration following AVP-ir axonal scaffolds (Figure 3(A1,A2), white arrows). Moreover, it was surprising to observe that there were at least two populations of AVP neurons within the region of the hypothalamic bed nucleus of stria terminalis (BNST-Hy). One population was located in the rostral part, with small somata and weak AVP immunostaining (Figure 3(B1,B2), indicated by black arrowheads), and another population was posterior and at the border of the stria medullaris (sm) and the fornix (f). The latter were strongly immunoreactive to AVP (Figure 3(B1,B2), white arrows). A cell chain alongside the sm was also observed (Figure 3C inset, red arrows). The migratory connection between the SON and the BNST-Hy, concluded from serial section analysis, is synthesized in diagram C of Figure 3. The vasopressin neuronal population’s distribution and function in the BNST-Hy has been described, with ample variations among reports. For example, vasopressin in the BNST has been linked to both anxiogenic (anxiety-promoting) and anxiolytic (anxiety-reducing) effects, depending on the circumstances [39]. A previous study of our group reported that one subpopulation of vasopressin neurons in the BNST-Hy co-expressed VGAT, a GABAergic neuronal marker; however, a substantial portion of the cells there did not express this marker, which suggests the existence of distinct subpopulations among vasopressin-expressing neurons within the BNST-Hy [40].

When the brains were sliced with a tilted angle of -30 degrees with reference to the septo-temporal axis (Figure 4A, top right inset, red line), the immunostaining in serial sections provided a fresh view regarding the anatomical organization of the PVN^AVP^ in adult rats. This figure shows examples from a 12-month-old male rat. An impressive AVP-ir cell chain alongside a main blood vessel was observed extending postero-laterally to the zona incerta (ZI) (Figure 4, red arrows). AVP-ir cells apparently dispersed from this chain, moving tangentially and reaching the nucleus basalis of Meynert (B) and the magnocellular nucleus of the lateral hypothalamus (MCLH) (Figure 4A, black and white arrows). The MCLH is a group of large cells located at the lateral border of the lateral hypothalamic area at the medial border of the internal capsule of the rat [41]. It is one of twenty six regions, zones, and nuclei constituting the motor lateral hypothalamus. Functionally, it belongs to the motor lateral hypothalamus of the subcortical motor system [41]. More examples are shown in Figure 4C–E. In Figure 4C, AVP-ir cell chains and dispersed neurons can be observed, with the amplified cell chain shown in (Figure 4D). In Figure 4E, tangential migrating AVP-ir cells in the medial amygdala, postero-dorsal subdivision, can be seen. A bipolar AVP cell alongside a blood vessel, with axonal swelling and apparent somal translocation, is indicated with a black arrow. The charting in Figure 4F synthesizes the observation from this cutting plane.

This pathway is further described in Figure 5, with the horizontal view at the interaural level of 2.20 mm, where the AVP-ir axon scaffolds’ guided cell migration can be appreciated. The PVN is delineated by dotted lines, and neurons migrated tangentially in a caudo-lateral direction toward the lateral hypothalamus (LH) and zona incerta (black arrows in Figure 5A,B). Strongly labelled AVP-ir somata were observed in the LH and ZI regions, with some neurons (red arrows in Figure 5B,C) clustered around blood vessels (BVs).

We recognize that the data presented thus far primarily imply the postnatal migration of AVP-ir neurons. The timing of this phenomenon—whether it takes place during early stages of life or persists into adulthood—remains unclear based on the aforementioned data.

The results mentioned above were all obtained from immunohistochemistry (IHC), mainly against vasopressin (except Figure 1(A2), with IHC against neurophysin II and doublecortin) in wild-type Wistar rat brain tissue. To further address this inquiry, we aim to supplement this report with evidence obtained from a parallel study involving CRISPR/Cas9-mediated targeted insertion of the IRES-Cre transgene into the 3′ untranslated region of the AVP gene in Sprague-Dawley rats. Two adult male AVP-IRES-Cre rats were injected in the PVN with a recombinant adeno-associated virus equipped with EF1a-DIO-mCherry. One month after viral injections, the rats were perfused, and their brains were analyzed as described below.

In Figure A1, we provide examples pertaining to the PVN viral vector injection in this transgenic AVP-IRES-Cre rat. In this instance, the lateral magnocellular division of the paraventricular nucleus of the hypothalamus (PVN_LM_, highlighted by the pink shadow region in Figure A1A and its inset) was targeted during viral infection of the same rat line. The rat brain was fixed one month after the viral injection. Figure A1B and its inset illustrate the targeted site, with numerous immunohistochemically labeled cells within the PVN_LM_^AV^ (outlined with dashed lines). The labeling was sparse yet precise, with no positive cells detected outside of the PVN_LM_ at this medio-lateral level. Within the PVN_LM_^AVP^, numerous neurons exhibiting tangential migration morphology (bipolar) were observed, as indicated by red arrows. Figure A1C depicts numerous immunohistochemically labeled cells forming cellular chains within the brain’s two main conducting systems, the *fornix* (f) and the *stria medullaris* (sm). The squared region was further magnified in Figure A1D, revealing labeled cells with tangential migrating morphology (red arrows). In Figure A1E, migrating cells are also present in another major conducting system, the *stria terminalis* (st), with migration morphology indicated by red arrows. It is worth comparing these mCherry IHC images in the transgenic rat line with Figure 2A–C, which show the IHC against AVP in wild-type rats; the very same morphological phenomena can be appreciated.

The presence of numerous neurons exhibiting tangential migration morphology within the PVN_LM_^AVP^ suggests active migration processes occurring in this nucleus of the adult rat brain following viral injection. Figure A1C–E, vividly illustrate the migration of labeled cells forming cellular chains within the *fornix* and *stria medullaris*, underscoring the ongoing neuronal migration processes during rat adulthood and emphasizing the dynamic nature of neuronal migration in adult rats subsequent to viral injection. It is worth noting that the *fornix*, *stria medullaris*, and *stria terminalis* constitute the main forebrain conducting systems with abundant axon bundles. The presence of neurons with migrating morphology within those conducting systems strongly suggest the transitory nature of the phenomenon, providing compelling evidence of neuron migration during adulthood.

**Figure 6:**
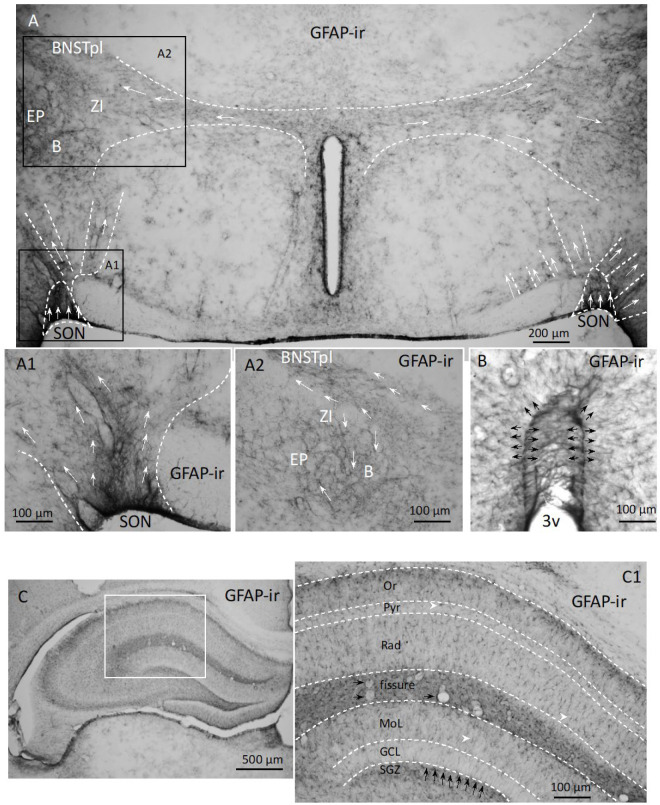
Glial fibrillar acidic protein (GFAP) immunostaining reveals a distinct feature of the GFAP-ir morphology, similar to the radial glial cells and their heterogenous distribution, resembling the migration routes observed with AVP IHC in the rat hypothalamus. ((**A**) the patterns of tangential migration routes described above are indicated with white arrows). (**A1**,**A2**): example of distribution of the GFAP-ir cells. (**B**) Tanycytes, a type of radial glial, in the periventricular region of the hypothalamus of the same reaction. (**C**,**C1**), GFAP labeling in hippocampus for reference. Note the similarity of the radial glial in the subgranular zone (SGZ), indicated by black arrows.

**Radial glial-like cell alignment reveals tangential migration paths.** Glial fibrillar acidic protein (GFAP) is the principal 8–9 nm intermediate filament in mature astrocytes and radial glial cells in the central nervous system [42,43]. It is known that astrocytes, through their interaction with neuroblasts and endothelial cells, play a key role in the genesis, migration, and integration of neurons in the adult brain [44,45]. We used anti-GFAP IHC to explore the astrocyte distribution in the hypothalamus. Surprisingly, we found distinct patterns of long GFAP-ir processes, which seemed to form bundles representing several migration routes (Figure 6(A–A2), indicated with white arrows). The labeling density was remarkably heterogeneous in the rat hypothalamus, and the observed routes coincided with the AVP-ir cells’ tangential migration routes described in Figure 1 and its supplemental figures. These cells appeared to resemble the tanycytes surrounding the third ventricle (3v) [19] in the hypothalamus (Figure 6B), as well as the glial cells found in the adult dentate gyrus [7] and subgranular zone (SGZ) of the hippocampal formation (Figure 6(C,C1)). These two types of glial cells are subtypes of the radial glial cells serving adult neuronal migration [43].

**Figure 7:**
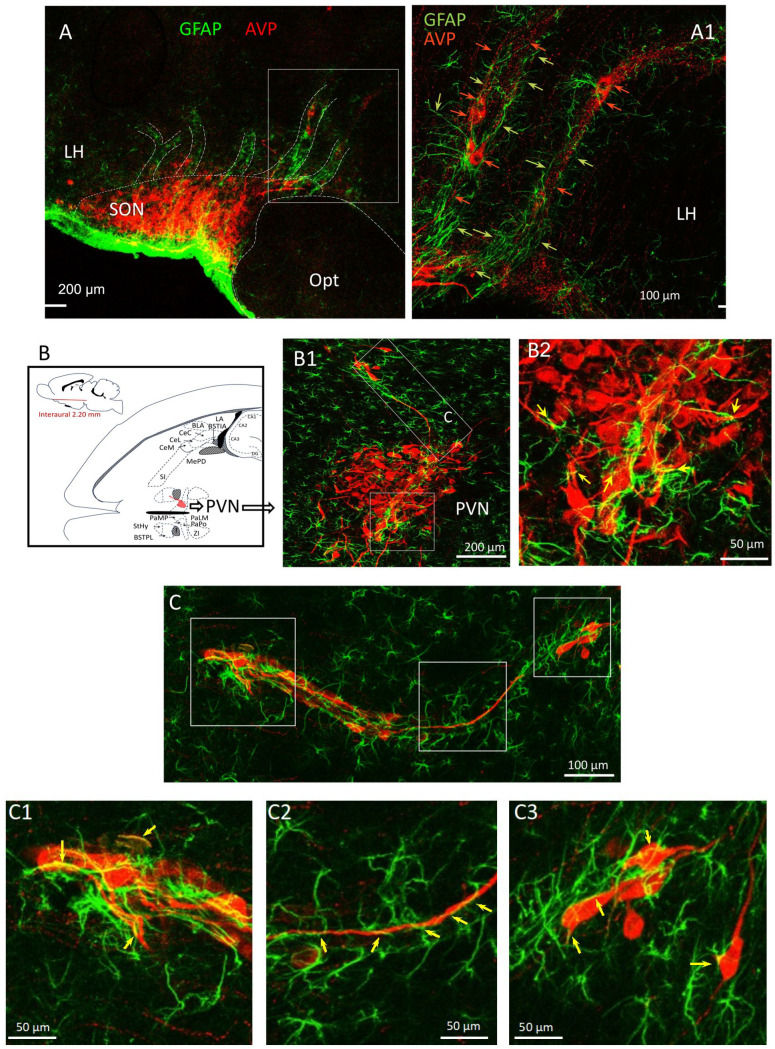
Double immunofluorescence reaction against AVP/GFAP revealed the intimate relationship between the AVP-ir dispersion, AVP neuronal chains, and the GFAP fibers. (**A**–**C**) GFAP-ir tunnel-like structures containing AVP-ir cells and axons. The phenomenon of AVP-ir axons “climbing” the GFAP-ir scaffolds is indicated with yellow arrows. (**C1**–**C3**) show the higher magnification of long GFAP-ir processes serving as migration scaffolds for AVP-ir soma and axons.

We further performed a double immunofluorescence reaction AVP/GFAP and found that all cell chains containing AVP-ir cells are closely associated with dense GFAP processes (Figure 7A–C). GFAP-ir long processes were seen intertwined with tangential-shaped AVP-ir cells and axons (Figure 7(C1–C3), yellow arrows) serving as migration scaffolds for AVP-ir soma and axons (Figure 7C).

**Figure 8:**
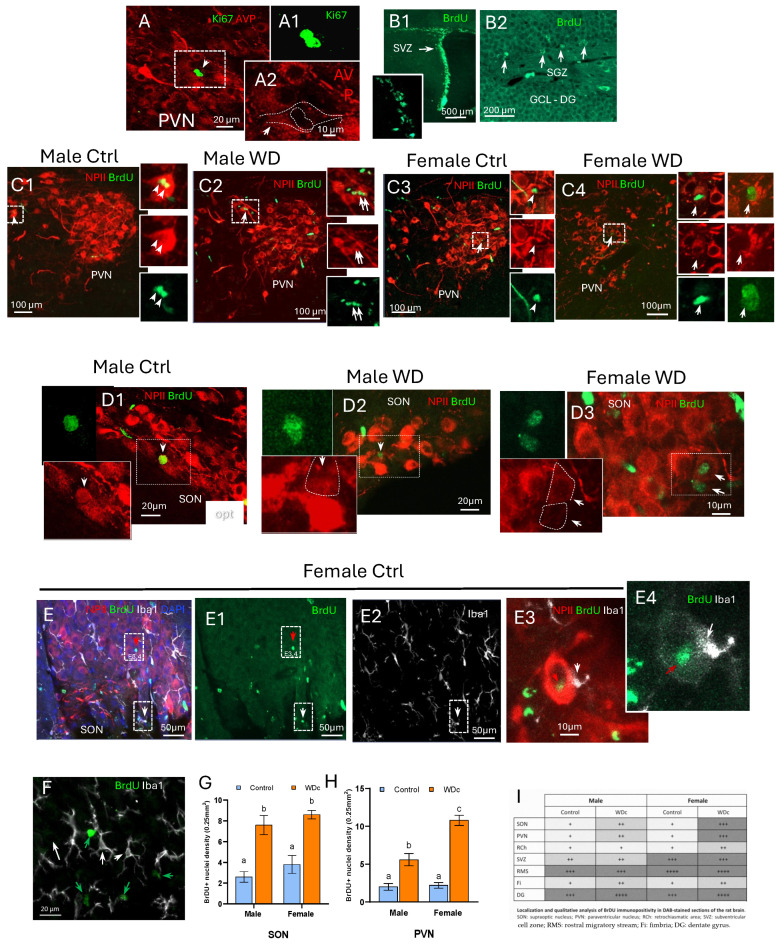
Neurogenesis occurs within the adult SON and PVN, and osmotic challenge significantly increases its rate. A panels show two Ki67-ir nuclei adjacent to each other (**A1**) within a swelled weakly AVP+ cell body (**A**,**A2**, dashed white line). We also used 5-bromo-2′-deoxyuridine (BrdU) injection followed by the immunostaining method to evaluate cell proliferation [47] in both euhydrated and chronic water-deprived (every other day, over 15 days) rats. B panels served as positive controls of the procedure, showing the BrdU-ir neurons within the canonical adult neurogenesis regions, (**B1**) subventricular zone (SVZ) and (**B2**) subgranular cell zone (SGZ) of the dentate gyrus. (**C**,**D**) panels show examples of BrdU co-labeling with neurophysin II, the carrier protein for AVP in the paraventricular (PVN) and supraoptic (SON) nuclei in both sexes, in euhydrated and water deprivation-challenged rats, respectively. BrdU-ir expression was also co-assessed with microglial marker Iba1. In (**E**), we show two BrdU-labeled nuclei (indicated with red arrow and white arrow inside two boxes). The nucleus indicated with a white arrow has a smaller diameter and overlaps with Iba1 expression (**E1**,**E2**), while the nucleus indicated with the red arrow has a larger diameter (7–8 µm). Panel (**E3**) shows an optical slice of 0.9 µm thickness at a different Z level of the same cell, where NPII and a branch of Iba1 fiber can be clear seen. (**E4**) shows the relationship of a microglial process making close contact to the BrdU-labeled cell. For clarity, the NPII signal was deactivated in this panel. Note that it seems the microglial plays a role in the newly born AVP MCN. Panel (**F**) shows that most of the BrdU-labeled nuclei are larger (7–8 µm, green arrows) than Iba1-labeled somata (white arrows); shading corresponding to the microglial nucleus is about 4 µm (NPII signal was deactivated in this panel). (**G**,**H**): statistical comparison of BrdU-ir nuclei in SON (**G**) and PVN (**H**), control vs. chronically water-deprived subjects. Bars with different lettering indicate significant differences at *p* < 0.01. (**I**): whole brain semi-quantitative assessment of BrdU-ir, control vs. WD. Serial sagittal brain sections were analyzed, and an index of positive nuclei (BrdU) in each 40× (0.45 mm^2^) field was assessed as follows: 1–4 nuclei (+); 5–9 nuclei (++); 10–15 nuclei (+++); more than 15 nuclei (++++). SON: hypothalamic supraoptic nucleus; PVN: hypothalamic paraventricular nucleus; RCh: retro-chiasmatic area; SVZ: subventricular cell zone; RMS: rostral migratory stream; Fi: fimbria; DG: dentate gyrus.

In order to demonstrate that cell proliferation occurs within the AVP magnocelular loci, we first used immunohistochemistry against the cell proliferation marker Ki67 protein [46] to test the neurogenic potential of AVP-MCN. In Figure 8, A panels, two Ki67-ir nuclei adjacent to each other (A1) within a swelled weakly AVP+ cell body are shown (A and A2, dashed white line). We also used 5-bromo-2’-deoxyuridine (BrdU) injection followed by the immunostaining method to evaluate cell proliferation [47] in both euhydrated and chronic water-deprived (every other day, over 15 days). B panels served as positive controls of the procedure; they show the BrdU-ir neurons within the canonical adult neurogenesis regions: B1, subventricular zone (SVZ) and B2, subgranular cell zone (SGZ) of the dentate gyrus. Figure 8C,D show examples of BrdU co-labeling with neurophysin II, the carrier protein for AVP in the paraventricular (PVN) and supraoptic (SON) nuclei in both sexes, in euhydrated and water deprivation-challenged rats, respectively. BrdU-ir expression was also co-assessed with microglial marker Iba1. In E panels, we show two BrdU-labeled nuclei (indicated with red arrow and white arrow inside two boxes). The nucleus indicated with the white arrow has a smaller diameter and overlapped with Iba1 expression (E1 and E2), while the nucleus indicated with the red arrow has a larger diameter (7–8 µm). Panel E3 is an optical slice of 0.9 µm thickness at a different Z level of the same cell, where NPII and a branch of Iba1 fiber can be clear seen. E4 shows the relationship of the Iba1 fiber with the BrdU-ir nucleus while the NPII soma signal was deactivated. It seems the microglia plays a role in the newly born AVP MCN. F panels show several complete microglial cells, indicating that the Iba1-ir nuclei size (white arrows) is of smaller diameter (4 µm) compared with most of the BrdU-labeled nuclei (7–8 µm, green arrows).

With the observations mentioned above, we then asked if neurogenesis could be modified as an allostatic mechanism for stress adaptation. This hypothesis was assessed by exposing adult rats to intermittent water deprivation (see materials and methods for detail) and injecting daily three doses of the thymidine analogue BrdU for 15 days. The same procedure of dehydration without BrdU injection was performed in order to study the possibility of BrdU interference with cell proliferation. Figure 8G,H show statistical comparisons of BrdU-ir nuclei in SON (G) and PVN (H), in control vs. chronically water-deprived subjects. The two-way ANOVA of SON revealed an effect of dehydration (F(1,16) = 47.54, *p* < 0.001) as the source of variation. There was no effect of sex (*p* > 0.1) nor interaction between factors (*p* > 0.5). In SON, multiple comparisons of groups revealed significant differences between male controls and male WD animals (*p* > 0.0001), between male controls and female WD (*p* < 0.0001), and between female controls and female WD (*p* < 0.01). In the case of PVN, differences were noted between male controls and male WD (*p* < 0.01), between male controls and female WD (*p* < 0.001), and between male WD and female controls (*p* < 0.001). In this case, an effect of sex as a difference between male WD and female WD (*p* < 0.001) was detected by statistical analysis. Female controls vs. female WD also showed differences (*p* < 0.0001) in the number of BrdU-positive nuclei. Different letters indicate differences between experimental groups. In congruence with this sexually dimorphic effect, it has been reported that estradiol can induce increased cell proliferation in the arcuate and dorsomedial hypothalamus [48]. Figure 8I depicts a whole brain semi-qualitative assessment of BrdU-ir, control vs. WD, in different brain areas where neurogenesis has been reported and the effect of WD on the BrdU-ir neuron density. As observed in the table, WD increased the number of BrdU + nuclei in the SON and PVN.

**Figure 9:**
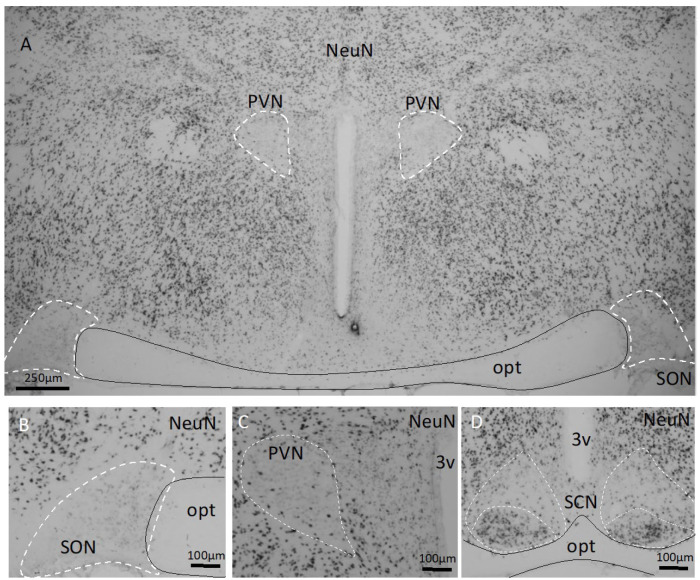
Absence of NeuN, a mature neuron marker, in the SON^AVP^ and PVN^AVP^. NeuN, a mature neuron marker, is widely expressed in the rat hypothalamus (**A**). However, both supraoptic (SON, (**A**,**B**)) and paraventricular (PVN, (**A**,**C**)) lack NeuN-expressing nuclei. In the suprachiasmatic nucleus (**D**), the ventral subdivision, which is mainly occupied by vasoactive intestinal polypeptide (VIP)-expressing neurons, shows high expression of NeuN, and the dorsal part, which is mainly occupied by AVP-expressing neurons, lacks NeuN expression. opt: optical tract; 3v: third ventricle. Black line outlines the optic tract and the white dashed lines outline the neuronal nuclei.

**Absence of NeuN, a mature neuron marker, from the SON and PVN in adult rats**. NeuN is a commonly used marker for postmitotic neurons. We performed immunostaining with this marker, aiming to assess the neuronal density of the hypothalamus. Surprisingly, in the magnocellular PVN and SON nuclei, there was a general lack of NeuN expression (Figure 9). In the suprachiasmatic nucleus, the ventral subdivision, which is mainly occupied by the vasoactive intestinal polypeptide (VIP)-expressing neurons [49], showed a high expression of NeuN, while the dorsal part, mainly occupied by AVP-expressing neurons [40], lacked NeuN expression.

## 3. Discussion

The PVN and SON nuclei of the hypothalamus are known for their role in the integration of hormonal, neuroendocrine, and behavioral responses as well as for being central hubs for adaptation to stress [50,51,52,53]. Animal studies have shown that exposure to stressors during the perinatal period leads to the increased metabolic activity of arginine-vasopressin magnocellular neurons, leading to increased volume and to a robustness in their dendritic branching [54].

Over the past fifteen years, we carried out a series of systems studies on the AVP-MCN ascending projection system in adult and older rats (12–18 months), as summarized in [28]. Connections between hypothalamic AVP-MCNs and other brain areas involved in stress coping and emotional/motivational behaviors were demonstrated through the employment of techniques such as juxtacellular labeling and tract-tracing methods combined with brain serial sectioning in cutting planes at coronal, sagittal, septo-temporal oblique, and horizontal orientations. Histochemical reaction against neurobiotin, immunohistochemistry (IHC) against antigens of interest, and systematic neuroanatomical analyses were performed [51,52,53,55,56,57,58]. During these studies, a collateral observation was repeatedly made: AVP immunoreactive (AVP-ir) magnocellular neurons seemed to be dispersed from the above-mentioned loci to adjacent subcortical regions. The lateral hypothalamus (LH), zona incerta (ZI), and latero-posterior division of the bed nucleus of the stria terminalis (BNSTlp) were observed as the main regions hosting these dispersed large AVP-ir neurons (DNs), but they were also seen in more distant subcortical regions, including the entopeduncular nucleus (EP), nucleus basalis of Meynert (B), and postero-dorsal division of medial amygdala (MEApd). The dispersed neurons were observed to be adjacent to blood vessels and AVP-ir fibers in scaffolds, some forming cords of AVP-ir cells. These chains were visualized first with AVP IHC, with AVP-ir cell bodies within it. The phenomenon was also observed in healthy older rats (18 months). These observations collectively suggested the occurrence of adult neuronal migration, raising the questions of the mechanism of their replenishment and potential generation via adult neurogenesis within the major AVP-MCN loci [8].

In the present study, we provide evidence on the neurogenesis and migration potential of AVP-MCNs in adult rats. Through systematic neuroanatomical analysis, we conclude that an important population of AVP-expressing neurons from the hypothalamic supraoptic (SON) and paraventricular (PVN) nuclei may continuously migrate to adjacent subcortical regions during adult life along streams of radial glia-like cells and processes and axon scaffolds. The recipient areas appear to include the lateral hypothalamus, zona incerta, bed nucleus of stria terminalis hypothalamic lateral posterior division, nucleus basalis of Meynert, magnocellular nucleus of the lateral hypothalamus (MCLH), and entopeduncular nucleus. In adult rat brain tissue, we could frequently observe such putative tangentially migrated AVP+ cells, which appear to be following AVP+ longitudinal tunnel-like bundles and matrices, which were outlined by GFAP+ radial glial-like fibers.

The process in which blood vessels guide neuronal migration has been reported to occur with adult neuroblasts as scaffolds [59]. These neuronal precursors are guided by several factors, originating in the vasculature, that use tyrosine kinase-associated receptors to activate migration. Examples of this are the presence of vascular brain-derived neurotrophic factor, BDNF, which is trapped by glia following its production by vascular cells to control the stationary/migratory phase of neuroblasts along the rostral migratory stream [60], or the vascular endothelial growth factor (VEGF), which regulates glial processes to orient and reorganize the vascular scaffolding as well as to act as a chemoattractant to neuronal progenitors [61]. These observations are of interest, as there are studies showing that magnocellular neurons are able to express VEFG [62] and that vasopressin is able to induce the production of VEGF via the stimulation of V1aR in endothelial cells [63].

We are aware of the limitation of the first finding, drawn based on our anatomical observation of serial sections from wild-type animals and the subsequent analysis. The conclusions solely based on these data shall be only intuitive. The physiological meaning of this phenomenon, i.e., adult AVP expressing MCN to disperse to other subcortical areas is still unclear; however, it raises important questions regarding whether the hypothalamic AVP system plays a role(s) in adult brain neural protection. For instance, in a study using in situ hybridization in human tissues, Swaab and colleagues reported that the amount of AVP mRNA in the hypothalamic suprachiasmatic nucleus of Alzheimer’s disease (AD) patients showed three-fold decrease vs. controls [64]. In another study, the authors report that patients with AD have decreased response to hyperosmolarity challenge and are prone to dehydration [65]. A recent study [66,67] reported intriguing multiomic data, describing the spatial transcriptome, proteome, phosphoproteome, and lipidome from the hypothalamic supraoptic nucleus. These studies indicate that there are four groups with distinct genetic features within the SON that are all related to AVP MCNs, offering new insight on possibilities of other AVP cell types within the SON. These cell types can be considered as candidates to study the mechanisms involved in regulating those neurons under physiological and pathological contexts [67].

Regarding the neurogenesis in the adult hypothalamus, one of the first studies that suggested that the hypothalamus could bear neurogenic potential was published almost twenty years ago, where the authors showed the presence of cells with immunohistochemical and electrophysiological characteristics of immature neurons in tissue cultures derived from the adult rat hypothalamus [68]; a subsequent study showed that the adult rat hypothalamus cultured neurogenic precursors had the capacity to generate neuropeptide-expressing neurons [35]. Interestingly a new population of radial glia-like neural stem cells has been recently reported in the postnatal hypothalamus, where Irx3 and Irx5 transcription factors modulate the neurogenesis of postnatally generated leptin-sensing neurons [69]. A recent comprehensive transcriptomic study has reported a change of 2247 RNA transcripts, including Ephrin receptor 6, a gene related to adult neurogenesis, in the SON of Wistar Hannover rats’ SON AVP MCNs, after 3 days of water deprivation [70].

Furthermore, the presence of DCX in AVP immunopositive neurons and the absence of the mature neuron marker NeuN suggest that a significant portion of the population are neuroblasts that have the ability to migrate, as DCX is a known marker of cell proliferation as well as migration [71,72]. The lack of expression of NeuN in the magnocellular PVN and SON nuclei and a segment of the suprachiasmatic nucleus where the majority of these neurons express vasopressin, suggests a transcriptomic identity that is different to the adjacent hypothalamic nuclei. It has been reported that some neuronal populations express low levels of NeuN [73], recently identified as an epitope of Rbfox3, an alternative splicing regulator [74]. Interestingly, many of the regions identified as not expressing NeuN are known to exhibit postnatal neurogenesis, i.e., the subventricular zone [5,75], the hippocampal subgranular zone of the dentate gyrus [76], the cerebellum [77], and the substantia nigra [78]. A number of other studies have shown that in local pathophysiological situations (disease/lesions), there is a reduction in the expression of NeuN without implying cellular death, but there is a loss of immunoreactivity [79]. For instance, after axotomy of the facial nerve, there is a reduction of NeuN immunoactivity in facial nucleus neurons [80]; after stroke, there is a reduction in neurons expressing NeuN in the penumbra and ischemic areas [81], and a re-localization of NeuN to the cytoplasm of HIV-infected neurons has been reported [82]. It has also been reported that a decreased expression of NeuN immunoactivity in hippocampal neurons is linked with exposure to neurotoxins [83] and a temporary loss of NeuN immunoreactivity in hippocampal neurons after radiation [84]. The above-mentioned studies suggest that no or low NeuN expression may be intimately linked with neuronal renewal mechanisms conserved to face brain homeostatic challenges and adaptation.

The field of neuroscience has both a ‘dogmatic phase’ and a ‘post-dogmatic phase’ with respect to the adult neurogenesis concept. Previously, observations of the apparent generation of neurons in the adult brain were simply dismissed. Following the discovery of the two major crypts of mitotically active neuronal precursors in the adult brain in the SVZ and the SGZ, the general concept of adult neurogenesis was subsequently embraced, but curiously, only for these two regions of the brain. The ‘non adult neurogenesis dogma’ appears to hold for the rest of the brain, even with these two exceptions. For example, the clear demonstration by Swaab and colleagues of adult neurogenesis in the vasopressin- and oxytocin-containing nuclei in pigs [29] has been quoted, as far as we are aware, only twice, until our report here.

There are two major findings recorded in our manuscript. The first is that adult AVPMNNs are capable of neurogenesis. While this has been suggested previously, it has not been unambiguously demonstrated using a combination of neurogenetic and peptide hormone markers until now. A second piece of the AVPMNN puzzle is provided by the use of AVP-promoter GFP-expressing rats, which now reveal the architectural dynamics of how new neurons are dispersed to extra-SON/PVN locations. The combination of proliferation and dispersion puts a new perspective on the ability of the SON/PVN to simultaneously regulate the neurohypophyseal hormonal system to correct hydromineral imbalance, and simultaneously provide new AVPMNNs at extrahypothalamic locations for stress adaptation.

## 4. Materials and Methods

### 4.1. Animals

This study was performed using thirty-four Wistar rats. All animal procedures were approved by the local bioethical committee. Rats were housed in plexiglass cages (2–3 per cage) attached to an air recycler device, with controlled humidity, temperature (22–25 °C), and filtered air ventilation, under controlled illumination (12:12 h light/dark photocycles). Food and water were provided ad libitum unless indicated otherwise.

### 4.2. Experimental Design

The study was divided in two parts. In the first part, systematic analysis and documentation of the patterns that some AVP neurons dispersed from the SON and PVN were made by anatomical examination of permanently mounted serial section samples with coronal, sagittal, horizontal, and septo-temporal oblique orientations, which were accumulated from our previous studies. The ethical statements, animal experiment licenses, and experimental details of those studies can be found elsewhere [51,52,53,55,56,57,58,85].

The second part concerned neurogenesis detection and demonstration using a 5′-bromo-2′-deoxyuridine (BrdU) protocol [11] and immunohistochemistry (IHC) against cell proliferation markers BrdU and Ki67 as well as migrating neuroblast marker doublecortin (DCX), and their relationship with GFAP and AVP expression. This part was performed under the license FM-CIE-079-2020.

### 4.3. Chemicals

Chemicals were obtained from Sigma–Aldrich, St. Louis, MO, USA, if not indicated otherwise. Sources of primary antibodies and their dilutions are depicted in Table 1.

### 4.4. Experimental Treatment of Water Deprivation (WD) and BrdU Injection

Twenty-four rats (250 +/−20 g, 12 per each sex) were used in this experiment, which consisted of two protocols. Rats were divided into two groups: controls, n = 6 × 2 (male and female) and water deprivation (WD), n = 6 × 2 (sexes). WD subjects were restricted from water intake every other 24 h. For protocol A, n = 4, intraperitoneal injection of the thymidine analog 5-bromo-2-deoxiurydine (BrdU, Sigma Aldrich) diluted in NaCl 0.9% (10 mg/mL) was given to each experimental subject for 14 days, with daily doses of 50 mg/kg body weight fractionated in 3 injections per day. Protocol B consisted of the same control and water deprivation treatment but without BrdU injection (n = 2 per group, N = 8) for Ki-67, GFAP, and DCX immunolabeling vs. AVP-ir.

### 4.5. Immunohistochemistry

Rats were deeply anesthetized with an overdose of sodium pentobarbital (63 mg/kg, Sedalpharma, Mexico) and perfused trans-aortically with 0.9% saline followed by cold fixative containing 4% paraformaldehyde in 0.1 M sodium phosphate buffer (PB, pH 7.4) plus 15% *v*/*v* saturated picric acid for 15 min (referred as Somogyi fixative [86]). Brains were immediately removed, blocked, then thoroughly rinsed with PB. Immunohistochemical reactions (IHC) were performed on the same day as perfusion/fixation and sectioning (Note: this timing scheme gave us remarkably better AVP labeling) using the immunoperoxidase (for single antigen labeling) and immunofluorescence (multi-antigen labeling) methods. The IHC procedure can be found elsewhere [51,52,53,55,56,57,58,85]. Briefly, brains were sectioned using a Leica VT 1000S vibratome, at 70 µm thickness, soon after perfusion/fixation (15 min) and thoroughly rinsed (until the yellow color of picric acid was cleared). The following sectioning planes were used: sagittal (n = 3), coronal (n = 3), semi-horizontal (30° to the horizontal plane, n = 2) and septo-temporal (between coronal and sagittal planes, 45° to both planes, n = 2). Freshly cut freely-floating sections (1 in 4) from different cutting planes were blocked with 20% normal donkey serum (NDS) in Tris-buffered (0.05 M, pH 7.4) saline (0.9%) plus 0.3% of Triton X-100 (TBST) for 1 h at room temperature and incubated with the primary antibodies listed in Table 1 in TBST plus 1% NDS over two nights at 4 °C with gentle shaking. For immunoperoxidase reaction, the conventional procedure were followed (detailed description can be found in [51,52,53,55,56,57,58,85]). For BrdU-IHC, sections were pretreated by incubating sections in 2N HCl for 30 min at 37 °C for DNA denaturalization. After this step, sections were rinsed twice with 0.1 M borate buffer (pH 8.5) followed by a rinse with phosphate buffer [87].

### 4.6. Transmission Electron Microscopy

The technical details of electron microscopy photomicrograph displayed in the inset of Figure 1 are reported elsewhere [55].

## Figures and Tables

**Table 1:** Primary antibodies used and their dilutions.

Antibody to	Host	Dilution	Source
[Arg8]-vasopressin	rabbit	1:2000	Peninsula laboratories international, T4563, Rheinstrasse, Switzerland
[Arg8]-vasopressin	rabbit	1:2000	Prof. R. M. Buijs, UNAM, Mexico
Neurophysin II	mouse	1:250	Prof. Harold Gainer, NINDS/NIH, Bethesda, MD, USA
Bromo-2-deoxyuridine (BrdU)	rat	1:100	Accurate Scientific, Carle Place, NY, USA OBT0030
Ki67	rabbit	1:250	Biocare Medical, Pacheco, CA, USA SP6
Glial fibrillary acidic protein (GFAP)	rabbit	1:1000	Biocare Medical, Pacheco, CA, USA, 901-040-031511
Neuronal nuclear antigen (NeuN)	mouse	1:2000	Millipore, Burlington, MA, USA, MAB377
Doublecortin (DCX)	goat	1:1000	Santa Cruz Biotechnology, Dallas, TX, USA, SC-8066
Iba1	rabbit	1:500	Biocare, Pacheco, CA, USA, CP 290A

## Data Availability

The original contributions presented in the study are included in the article; further inquiries can be directed to the corresponding authors.

## Appendix A

### Supplemental Methods

The transgenic rat strain, AVP-IRES2-Cre knock-in rats, was generated by CRISPR/Cas9-mediated targeted insertion of the IRES2-Cre transgene in the 3′ untranslated region of the AVP gene in Sprague-Dawley rats [88]. This strain allows us to specifically target distinct AVP neurons via stereotaxic injections of Cre-dependent viral vectors, to express any genes of interest. Two adult AVP-IRES2-Cre knock-in transgenic rats (one female and one male) were injected with the viral vector one month before the perfusion-fixation. Fixed brain tissues were sliced in the sagittal plane with a Leica vibratome (VT1000), and serial sections were processed and immunostained with a goat anti-mCherry antibody (Goat tdTomato Polyclonal Antibody-AEG42798.1, MBS448092, MYBIORESOURCE, San Diego, CA, USA). This antibody (MBS448092) is specific for tdTomato and mCherry proteins). Samples were incubated with a primary antibody (1:2000) over two nights, at 4 °C, with gentle shaking, and then incubated with a secondary antibody, rabbit anti-Goat IgG Antibody (H+L), biotinylated (BA-5000-1.5), 1:500 (Vector Laboratories, Newark, CA, USA) over night, at 4 °C (this procedure produces a good ultrastructure if examined under electron microscope). Finishing the secondary antibody incubation, samples were incubated with an avidin-biotin-peroxidase complex (ABC) using immunoperoxidase techniques (Vector Laboratories, Newark, CA, USA). DAB (3,3′-Diaminobenzidine, 0.05% in 0.1 M phosphate buffer, pH 7.4) was oxidized by hydrogen peroxide in a reaction typically catalyzed by horseradish peroxidase (HRP), which was linked to the B component of the ABC complex. The oxidized DAB forms a brown precipitate, at the location of the HRP, linked to the presence of mCherry, which was linked the vasopressin promotor. Samples were then examined under light microscopy.
